# Aristotle Meets Zeno: Psychophysiological Evidence

**DOI:** 10.1371/journal.pone.0168067

**Published:** 2016-12-29

**Authors:** Charalabos Papageorgiou, Xanthi Stachtea, Panos Papageorgiou, Antonio T. Alexandridis, Eleftheria Tsaltas, Elias Angelopoulos

**Affiliations:** 1 1st Department of Psychiatry, Eginition Hospital, Athens, Greece; 2 University Mental Health Research Institute (UMHRI), Athens, Greece; 3 Department of Electrical and Computer Engineering, University of Patras, Patras, Greece; Banner Alzheimer's Institute, UNITED STATES

## Abstract

This study, a tribute to Aristotle's 2400 years, used a juxtaposition of valid Aristotelian arguments to the paradoxes formulated by Zeno the Eleatic, in order to investigate the electrophysiological correlates of attentional and /or memory processing effects in the course of deductive reasoning. Participants undertook reasoning tasks based on visually presented arguments which were either (a) valid (Aristotelian) statements or (b) paradoxes. We compared brain activation patterns while participants maintained the premises / conclusions of either the valid statements or the paradoxes in working memory (WM). Event-related brain potentials (ERPs), specifically the P300 component of ERPs, were recorded during the WM phase, during which participants were required to draw a logical conclusion regarding the correctness of the valid syllogisms or the paradoxes. During the processing of paradoxes, results demonstrated a more positive event-related potential deflection (P300) across frontal regions, whereas processing of valid statements was associated with noticeable P300 amplitudes across parieto-occipital regions. These findings suggest that paradoxes mobilize frontal attention mechanisms, while valid deduction promotes parieto-occipital activity associated with attention and/or subsequent memory processing.

## Introduction

Reasoning ability is the vehicle of extrapolation based on the available information, even when is incomplete. Deductive reasoning allows the formulation of relationships between premises and potential conclusions and is therefore a hallmark of higher cognition. Nevertheless, the brain mechanisms underlying it remain obscure [1,2].

Logical reasoning is usually assessed by tasks in which participants are instructed to judge as quickly as possible the logical validity of syllogisms consisting of two statements (the premises) and a conclusion. An example of the two premises of a syllogism would be: “All men are mortal”; “All Athenians are men”. Aristotle concluded that these premises imply with absolute certainty that “All Athenians are mortal” [3].

In juxtaposition to this, about 2500 years ago, Zeno of Elea, student of Parmenides, introduced the paradoxes in which he defends the value of the Eleatic philosophy by attempting to prove that change (motion) and plurality are impossible (Plato *Parmenides 128 d*) [4]. In the paradoxes Zeno utilized the method of indirect proof or *reductio ad absurdum*, which involves temporarily assuming some thesis which the orator is in fact opposed to, and then attempting to deduce an absurd conclusion or a contradiction, thereby undermining the original assumption.

In the Parallel Lives [5] Plutarch remarks: ‘Pericles was also a pupil of Zeno the Eleatic, who discoursed on the natural world, like Parmenides, and perfected a species of refutative catch which was sure to bring an opponent to grief; as Timon of Phlius expressed it: "His was a tongue that could argue both ways with a fury resistless, Zeno's; assailer of all things". Indeed, Zeno’s paradoxes have ever since intrigued philosophers and mathematicians stimulating subsequent research [6,7], as this reasoning can be viewed as a form of cognitive illusion. It has been commented that it is fortunate that such cognitive illusions violate the norms of rational thought only in the context of philosophical speculation [7,8].

Current approaches to the study of reasoning have introduced specific models of encoding and reasoning mechanisms [9–11]. The proponents of formal rules claim that reasoning problems are solved on the basis of a set of inference rules, which implies that reasoning is mainly a linguistic process [12,13]. In contrast, mental model theorists propose that reasoning stems from mental sets of the situation presented by the premises: those sets are considered to be spatial in nature [10,14]. It follows that reasoning functions should be subserved by regions specializing in visualization, such as the right hemisphere parieto-occipital areas which are involved in visuospatial processing. Evans [9,15] and Goel [16] attempted to reconcile the two views presented above by coining the theory of dual-process reasoning, which postulates two distinct reasoning systems subserved by separate neurobiological substrates [9,15,16]. System 1 is described as a rapid, parallel and automatic process mainly located in the fronto-temporal area, while System 2 is a slow, serial process involving working memory and parieto-occipital areas.

Increasing effort has recently been invested towards the elucidation of the neural substrate of reasoning. This has been facilitated by technological advances, including brain imaging techniques and event related potentials.

Brain imaging studies of reasoning have produced a large body of evidence which, however, is characterised by great cross-study variability in brain responses. The observed variance has been attributed to a variety of sources ranging from the modes of reasoning adopted by various studies to differences in their experimental design [17–19]. The basic traits emerging from the results of the imaging studies suggest that familiar, conceptually coherent material engages a left lateralized fronto-temporal system associated with conceptual and linguistic operations. It has been proposed that this corresponds to a heuristic system [20]. In contrast, novel, incoherent material appears to engage a bilateral parietal visuospatial system [9,21]. A recent meta-analysis based on 28 imaging studies published between 1997 and 2010 and studying a total of 382 participants concluded that, during reasoning, there is a consistent tendency of mainly left lateralized activations, with other subsystems being recruited depending on the nature of the task (propositional, categorical, or relational reasoning) [22]. Recently Oaksford [23] challenged these conclusions, commenting that ‘First, the main function of the core brain region identified is most likely elaborative, defeasible reasoning not deductive reasoning. Second, the subtraction methodology and the meta-analytic approach may remove all traces of content specific System 1 processes thought to underpin much human reasoning. Third, interpreting the function of the brain regions activated by a task depends on theories of the function that a task engages’.

In addition to the brain imaging studies, the neural substrate of deductive reasoning has also been investigated by event related potential (ERPs) studies [24–26]. The high resolution properties of ERPs and the fact that several evoked waveforms have a well documented association with specific information processing functions renders this technique particularly useful in the study of brain activity during reasoning [27,28].

In a syllogistic reasoning study investigating 14 healthy adult subjects Qiu et al. [29] reported that a valid syllogistic reasoning task elicited a greater positive ERP deflection corresponding to the P3b component than an invalid task and a baseline task after the onset of the minor premise. Dipole source analysis indicated that this component was localized in the occipito-temporal area, which is possibly related to visual premise processing.

Valid and invalid reasoning tasks compared to a baseline task elicited both a significantly higher negative waveform between 600 and 700 ms, and a positive waveform between 2500 and 3000 ms. Dipole source analysis revealed a medial frontal cortex/anterior cingulate cortex localization for the negative waveform difference, and a strong activity in the right frontal scalp regions for the positive one. The authors adhered to the views of Knauff et al. [30] and Ruff et al. [31] who reported that reasoners utilise spatially organized mental models to solve deductive problems.

Bonnefond et al. [32] examined ERPs evoked by conclusions of the most commonly used concepts in logic, *Modus Ponens* (if P implies Q and P is true, therefore Q must be true). They found that valid experimental conditions, in contrast to invalid ones, were associated with pronounced P3b. Based on the assumption that the P3b component reflects information processing satisfying expectations, they interpreted their results to mean that participants had already drawn the *Modus Ponens* inference based on the major premise presented. They therefore argued that conditional inferences, particularly *Modus Ponens* ones, are drawn spontaneously. In a more recent study Bonnefond et al. [33] recorded ERPs obtained during a conditional inference task with either many or few disabler conditionals. They found that many disabler conditionals produced a more pronounced N200 waveform and a decreased P3b. They concluded that thematic content does not necessarily alter subjects’ inferences.

Taking into consideration the findings presented above, we elected to focus on the auditory P300 component while participants were required to draw a logical conclusion regarding the correctness of valid syllogisms or paradoxes. The P300 is produced by a distributed network of brain processes associated with attention and memory operations. The functional and neuropsychological data regarding the P300 potentials suggest two distinct components reflecting two different processes within the human brain. Specifically, the P3a, with frontal location, has been linked to the initial allocation of attention, while the P3b component has been related to activation of a posterior network when the neuronal model of perceived stimulation is compared with the attentional and /or working memory operation [34,35].

In the light of the above we hypothesized that electrophysiological brain activity, as reflected by the P300 component, would be useful in identifying differences between valid deductive reasoning and paradoxical reasoning, presented in a way designed to engage working memory (WM) operation. WM refers to the ability to retain information ‘on line’ in order to facilitate an ongoing task [36,37]. It forms a substrate for complex cognitive functions including planning, problem solving, decision making and reasoning [38–40].

In brief, using a relatively homogenous sample of adults and task forms designed to engage WM operation, the present study was designed to determine whether valid deductive reasoning on the one hand and paradoxical reasoning on the other, will elicit different patterns of electrophysiological activity, as reflected by P300.

## Methods

### Participants

This study was approved by the Ethics committee of University Mental Health Research Institute (UMHRI). A number of fifty-one healthy subjects (aged 33.9 years in average, standard deviation: 9.2; 28 males) participated in the experiment. All participants gave written consent, after being extensively informed about the procedure. They all had normal vision and no one had neurological or psychiatric history.

### Stimuli and procedures

#### Stimuli

The experiment was designed to juxtapose two mental functions: processing of syllogisms characterized as “valid” vs processing of “paradoxical” reasoning. This was done by exposure of the participants to two arrays of statements, one containing 39 valid syllogisms, the other 39 paradoxes (S1 File). The order of presenting the valid and paradoxical sections was counterbalanced across participants. The full arrays of valid and paradoxical statements used are available in a supplemental file submitted to the journal. Two indicative examples follow: the valid array included statements of the following type: “*All men are animals*. *All animals are mortal*. *Hence*, *all men are mortal*.” [41]. The paradox array consisted of statements of the following type: “*A moving arrow occupies a certain space at each instant*. *But*, *when an object occupies a specific space*, *it is motionless*. *Therefore*, *the arrow cannot simultaneously move and be motionless*.” (Aristotle, Physics VI: 9, 239b5) [3].

#### Behavioural procedure

Each participant was seated comfortably 1 m away from a computer monitor in an electromagnetically shielded room. He / she was verbally instructed through the intercom to read carefully each statement which would appear in the monitor, followed by the question “Right OR Wrong?” and state verbally, after two presentations of a warning sound (a) whether the statement was right or wrong and (b) how certain, on a scale of 0 (not at all certain) to 100 (absolutely certain) he/she was of the answer. This verbal instruction was followed by two examples of valid and two of paradoxical statements as a training exercise ensuring that the participant had fully comprehended the task. After 2 min. of rest he/she was instructed to initiate the formal experimental session by pressing the SPACE bar.

Once the participant initiated the procedure, the sequence of statements forming a valid or paradoxical reasoning proposition was presented on the screen. Each statement remained on the screen for a duration determined by the number of digits included in the sentence (see Table 1) and then was replaced by a blank screen for a period of 1000 ms. This was followed by a 500 Hz auditory warning stimulus of 65 dB and 100 ms duration, which was repeated after 900ms. The participant’s verbal response and degree of confidence in the answer to each statement was recorded by an observer seated outside the experimental chamber.

**Table 1:** Precise sequence of phases of the performed experiments.

Sequence of actions	Duration of actions
Valid or paradox sentence (visual presentation)	Duration according to the numbers of the letters in the sentences e.g. a sentence involving 92 letters presented 11,04sec
EEG recording	1000ms
Warning stimulus	100ms
ERP recording	1sec
Warning stimulus repetition	100ms
Response onset	Within 5sec
Period between response completion and onset of next sentence presentation	4–9sec

The onset of the next statement followed completion of the previous verbal responses after a variable interval of 4 to 9 sec in order to avoid habituation with temporal test sequences.

#### Electrophysiological procedure

**Fig 1:**
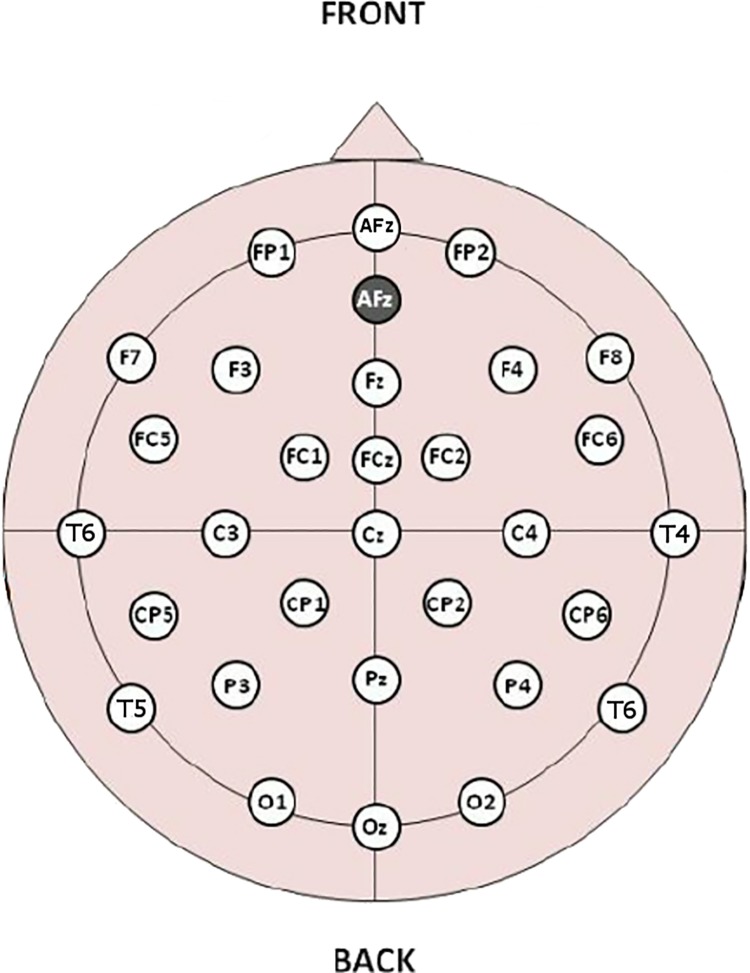
Map showing the position of the ERPs' electrodes.

Before entering the electromagnetically shielded test room the participant was fitted with a cap equipped with 30 scalp electrodes and 2 reference potential electrodes, each attached to an ear lobe (see details of electrode placements in the section on Experimental setup and Recordings and Fig 1). Through these electrodes EEG was recorded for 1000msec before the first warning stimulus (EEG) and for 1000msec after that (ERP).

A summary of the behavioural and electrophysiological events sequence of the procedure is presented in Table 1.

### Experimental setup and recordings

A Faraday cage was used in order to eliminate any electromagnetic interference that could affect the measurements; the attenuation of the mean field was more than 30dB. 30 scalp Ag/AgCl electrodes were employed to record the electroencephalographic (EEG) activity in accordance with the International 10–20 system of electroencephalography [42]. A map of the electrode constellation is shown in in Fig 1. Two electrodes, each attached to an ear lobe, served for obtaining the reference potential.

Recordings with EEG higher than 75μV were excluded. Electrode resistance was kept constantly below 5kΩ. The bandwidth of the amplifiers was 0.05–35Hz, in order to avoid interference due to the power supply network's signal, which is at 50Hz. Eye movements were recorded by means of an electro-oculogram (EOG). The brain signals are amplified by a Braintronics DIFF/ISO-1032 amplifier before entering a 32-bit analogue to digital converter (NI SCB-68) which has a GPIB output. The digitized signal comprised an input for a Data Acquisition Card. The PC with the DAQ Card runs a LabView program for the recording of the signals, which can be monitored by an on-screen graphical representation. The evoked bio-potential signal was digitalized at a sampling rate of 1Khz. The signals were recorded for a 2000msec interval, namely 1000msec before the first warning stimulus (EEG) and 1000msec after that (ERP).

For each question and for each electrode separately, 2000 samples (expressed in μV) have been recorded in 2sec; evidently, the employed sample period was 1ms. For each question separately, we averaged the values of the EEG, namely the data acquired in the 100ms before the first sound stimulus.

**Fig 2:**
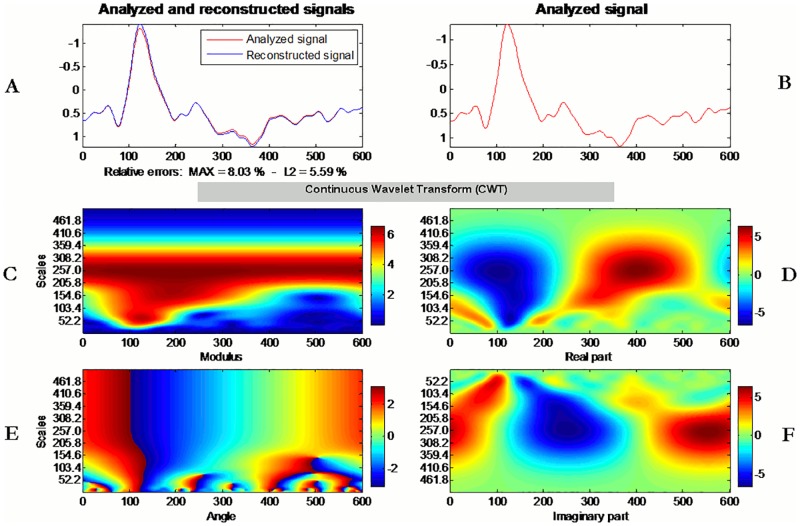
Mean ERP signal of lead P4, in the positive condition, is analyzed. Time frame displayed in figures is oriented to the time point of trigger onset to 600 ms after. Each coefficient in the scalograms represents a degree of correlation between the transformed wavelet and the analyzed signal. Warmer coloured regions indicate a stronger degree of correlation. (A) Analyzed and reconstructed ERP signals. (B) Depiction of analyzed ERP waveform. (C) Modulus of complex Morlet wavelet. (D) Real part of complex Morlet wavelet. P300 component can be identified in the scalogram. (E) and (F) visualize the angle and the imaginary part of the complex Morlet wavelet used in CWT.

We subtracted the obtained average from the initial signal, thus obtaining a translated version of the specific ERP recording. Thereafter, for the ERP detection, a Continuous Wavelet Transform (CWT) algorithm was developed, using EEGlab (Delorme & Makeig, 2004), running under Matlab^®^ 2013 (MathWorks, USA), along with the Wavelet Toolbox^™^ [43]. A detailed description of the procedure is provided in the Appendix. We considered the wavelet coefficients obtained by analyzing and reconstructing the evoked potential calculated via conventional averaging in each participant. A demonstration of the wavelet transform is visualized in the scalograms of Fig 2, where each coefficient represents a degree of correlation between the transformed wavelet and the analyzed signal.

Based on these coefficients, an appropriately scaled wavelet was chosen to match the P300 component. The wavelet was convolved with the EEG signals, only in the corresponding part of the signal where P300 component could be situated (240–500ms after the trigger onset), thus avoiding a false ERP detection. ERP peak values and corresponding latencies were extracted for each EEG channel of each participant, for each condition.

### Statistical analysis

#### Electrophysiological data

The amplitudes and latencies of the component P300 taken over the range of 220–500 ms, were subjected to pair t-test analysis. The P300 dataset is provided as S2 File.

The STATISTICA 10.0 for Windows (Statsoft Inc. Tulsa, OK, USA) software was used to assess the statistical significance of the observed differences between the Valid and Paradoxes measurements by means of a standard t-test for related samples (repeated measures). Due to the multiple comparisons the Bonferoni correction was applied to all p-levels.

#### Behavioural data

Behavioural responses were coded as follows: for the 39 valid statements “Right” was considered the correct answer whereas for the 39 paradoxes “wrong” was considered correct (responses coded as 0 and 1 for incorrect and correct responses respectively, and the total of correct responses is presented for each condition. Total accuracy per condition was expressed as the proportion of correct answers per 39 opportunities. The average confidence of each subject about their judgements is also presented in the form of a percentage.

## Results

### Electrophysiological results: Comparison of P300 amplitudes of the two conditions

#### Comparison of P300 amplitudes and latencies of the two groups

**Fig 3:**
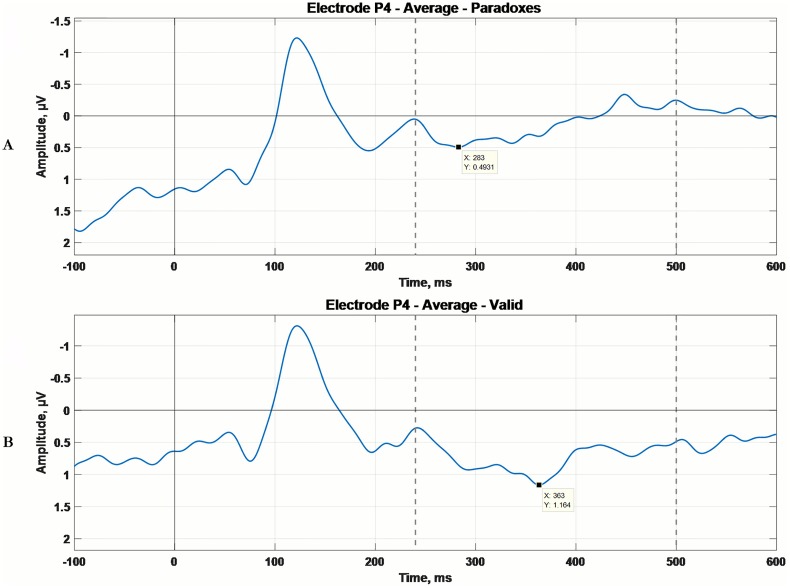
Grand average ERP waveforms at lead P4. A) Grand average ERP waveform in microvolts, for the Paradoxes group at lead P4. B) Grand average ERP waveform in microvolts, for the Valid group at lead P4.

In Fig 3 the grand average ERP waveforms for the two groups under investigation are shown, at lead P4.

Mean amplitudes, in microvolts, of P300 amplitude waveform for the two groups, at each lead are displayed in Table 2.

**Table 2:** Mean amplitudes, in microvolts, of P300 amplitude waveform for the two groups, at each lead.

		Valid	Paradoxes	p
		Mean±Std	Mean±Std	
1	F7	0,9745±2,28764	1,5715±1,75737	0,048
2	FC5	0,6606±1,27547	1,0264±1,35858	0,070
3	C3	0,7459±,87856	,9014±1,01409	0,207
4	CP1	1,7696±1,40759	1,6680±1,85782	0,690
5	P3	2,3184±1,56435	2,0566±1,56109	0,197
6	FPZ	1,1246±2,68769	2,3532±2,36961	0,001*
7	AFZ	0,8429±2,50338	1,9151±1,95241	0,000*
8	CZ	1,1239±1,23852	1,1260±1,00673	0,991
9	O1	3,7194±2,28322	3,1699±2,16089	0,011*
10	O2	3,7103±2,44903	2,8750±2,05637	0,001*
11	F8	0,9719±2,45147	2,1806±2,11570	0,000*
12	FC6	0,9914±1,39716	1,5628±1,27516	0,009*
13	T4	2,0694±1,37379	2,0001±1,34584	0,608
14	CP2	1,9742±1,44218	1,6357±1,36975	0,046
15	P4	2,4693±1,70568	1,9107±1,35425	0,004*
16	CP6	8,5886±5,79086	7,9981±5,66981	0,410
17	T6	2,9253±2,32218	2,3502±1,53026	0,034
18	F3	,7923±1,68613	1,2165±1,39349	0,080
19	FC1	,6617±1,34783	1,0199±1,17109	0,033
20	T3	1,7026±1,35377	1,5430±1,43469	0,463
21	CP5	1,6998±1,31621	1,5115±1,17666	0,284
22	T5	3,1095±1,95552	2,9423±2,07264	0,552
23	FP1	1,1952±2,57481	2,7082±2,39992	0,000*
24	FP2	1,1755±2,70988	2,7339±2,44529	0,000*
25	FZ	5,1805±2,91143	4,6739±2,92500	0,155
26	PZ	2,4464±1,75907	1,9989±1,42685	0,019
27	OZ	3,6355±2,42617	2,8700±2,03747	0,002
28	F4	,6716±1,69894	1,5514±1,48180	0,001
29	FC2	,8150±1,33921	1,3044±1,30180	0,021
30	C4	1,2406±1,41767	1,4213±1,60468	0,213

*p value statistically significant at 0.05 level by post hoc Bonferroni correction.

The Bonferroni post hoc procedure revealed that mean amplitude values at leads Fp1, Fpz, Fp2, AFz, F8, FC6 for valid deduction were significantly lower than mean amplitude values for paradoxes, while the opposite picture was obtained regarding the leads P4, O1 and O2. The latency waveforms for the two groups did not show any significant dissimilarity.

### Behavioural results: comparison of response accuracy and confidence between the two conditions

As seen in Table 3, mean correct responses in the valid condition (judgement = “right”) were higher than those noted in the paradoxes (judgement = “wrong”; 29.25 and 19.49 respectively). The difference was statistically significant (paired t-test t = 10.67, p = 0.00). Similarly, the self-reported confidence observed with respect to the valid condition as opposed to the paradoxes was significantly higher (means 82.14 and 77.43 respectively, paired t-test: t = 4.08, p = 0.00).

**Table 3:** Response accuracy and confidence for the Valid and Paradoxical conditions.

	**Mean**	**Std.Dv.**	**N**	**Diff.**	**t**	**df**	**p**	**-95%**	**+95%**
Score Valid	29,26	5,65							
Score Paradox	19,49	5,45	51	9,77	10,67	50	0,000	7,93	11,60
	**Mean**	**Std.Dv.**	**N**	**Diff.**	**t**	**df**	**p**	**-95%**	**+95%**
Confidence Valid	82,14	11,16							
Confidence Paradoxes	77,43	13,55	51	4,71	4,08	50	0,000	2,39	7,03

### Correlations between electrophysiological and behavioural data

When correct responses were examined separately in the valid syllogism condition, moderate but statistically significant positive correlations were noted between the correct responses and P300 in electrodes O1, O2, CP6, FZ, and OZ. Also, moderate but statistically significant negative correlations were observed in electrodes FC6 and F4. On the measure of self-reported confidence, there were two positive correlations found in electrodes FC5 and F3 and a negative correlation in electrode T4. In contrast, when correct responses were examined in the paradox condition, no correlations were noted between P300 and the corresponding correct responses or self-reported confidence. (Table 4).

**Table 4:** Correlations P300 Valid and Paradoxes with Score and Confidence.

	Score Valid	Confidence Valid	Score Paradoxes	Confidence Paradoxes
P300 FC5	-0,2252	0,2951	-0,0359	0,0288
	N = 51	N = 51	N = 51	N = 51
	p = 0,112	p = 0,036	p = 0,802	p = 0,841
P300 O1	0,2926	-0,2193	0,1435	0,0814
	N = 51	N = 51	N = 51	N = 51
	p = 0,037	p = 0,122	p = 0,315	p = 0,570
P300 O2	0,3143	-0,1753	0,0731	0,1232
	N = 51	N = 51	N = 51	N = 51
	p = 0,025	p = 0,218	p = 0,610	p = 0,389
P300 FC6	-0,3038	0,0834	-0,0551	-0,1153
	N = 51	N = 51	N = 51	N = 51
	p = 0,030	p = 0,561	p = 0,701	p = 0,420
P300 T4	0,1248	-0,3609	0,1006	-0,2079
	N = 51	N = 51	N = 51	N = 51
	p = 0,383	p = 0,009	p = 0,483	p = 0,143
P300 CP6	0,3328	-0,2327	0,2283	-0,2644
	N = 51	N = 51	N = 51	N = 51
	p = 0,017	p = 0,100	p = 0,107	p = 0,061
P300 F3	-0,1716	0,3135	0,1252	0,1394
	N = 51	N = 51	N = 51	N = 51
	p = 0,229	p = 0,025	p = 0,381	p = 0,329
P300 FZ	0,3891	-0,1676	0,1164	-0,1498
	N = 51	N = 51	N = 51	N = 51
	p = 0,005	p = 0,240	p = 0,416	p = 0,294
P300 OZ	0,2835	-0,1209	0,0594	0,0529
	N = 51	N = 51	N = 51	N = 51
	p = 0,044	p = 0,398	p = 0,679	p = 0,712
P300 F4	-0,3123	0,1232	0,0750	0,0364
	N = 51	N = 51	N = 51	N = 51
	p = 0,026	p = 0,389	p = 0,601	p = 0,800

## Discussion

During paradox deduction, our results demonstrated a more positive event-related potential deflection (P300) across frontal regions, specifically at leads Fp1, Fpz, Fp2, AFz, F8, FC6. In contrast, valid deduction was associated with noticeable P300 amplitudes across parieto-occipital regions, in particular at leads P4, O1 and O2. The significance of the observed differences concerning P300 amplitudes can be better understood if both psychophysiological and neurobiological aspects of this ERP component are taken into consideration. The P3 component of the event-related potentials is consistently related to attention, decision making and memory updating and therefore provides a valuable tool for the investigation of these processes in the human brain. It represents two distinct though strongly interrelated subcomponents P3a and P3b [35,44].

The suggested source of the P3a is the frontal lobe. Patients with frontal lesions demonstrate attenuated P3 amplitude at frontal sites, while their parietal response appears less affected [45–47]. These results are in line with recent neuroimaging and ERP studies demonstrating that activity of the frontal cortex can be related to detection of infrequent or alerting stimuli [48–51]. P3a has been associated with the initial reallocation of attention resulting from detection of stimulus attribute changes. This process follows original sensory processing and stimulus feature mismatch detection. The prefrontal cortex is required for cognitive organization in decision making under ambiguity [52,53] and also appears to play a key role in the cognitive control of conflict [54]. In this context it is worth noting a study by Goel et al. [55] who investigated 17 normal volunteers with fMRI while they engaged in a transitive inference task involving determinate and indeterminate relations. The results showed that the right ventrolateral prefrontal cortex (BA 47) is activated by the processing of indeterminate trials with no belief-bias cues, while left lateral prefrontal cortex (BA 45) is activated by the processing of indeterminate trials with belief-bias cues. The authors suggested that these results reflect an interplay between the tendency of the left PFC to overinterpret information [56] and the ability of the right PFC to moderate this overinterpretation by maintaining ambiguous or indeterminate mental representations of the situation at hand [57]. Essentially Goel et al. [55] claimed that right hemisphere contribution is twofold. On the one hand, it prevents the left hemisphere from premature pattern completion, hence inhibiting premature conclusions. On the other hand it maintains the incomplete pattern on line for further evaluation. Our results, which indicate increased P300 activity involving the frontal cortical regions during exposure to paradoxes, would appear to be in line with the above.

In contrast with the P3a, the P3b has a more posterior-parietal scalp distribution. There is ample evidence that the P3b component can be viewed as indexing voluntary attention, its amplitude reflecting the allocation of attentional resources [57,58]. This process should engage working memory operation, while the neuronal model of the stimulation is compared with the attentional trace of relevant information. Our results show an association of the P3b with valid arguments. This is in accordance with brain imaging observations situating reasoning-related activities in parietal and/or occipital regions of the brain, which are known to be engaged in tasks with visuospatial components [59]. Furthermore, our results are compatible with previous ERP studies, as long as they have focused on P3- like waveforms [29,32,33].

At this point the relationship between electrophysiological data and behavioural performance consisting of response accuracy and confidence in responses made must be discussed. In the valid statements condition, accuracy was positively correlated with P300 amplitudes located at occipital and parietal areas (O1, O2, Oz leads and CP6 lead respectively) which mediate bottom-up processing, as well as with prefrontal areas (Fz lead) mediating top down processing. Additionally, it was found that accuracy exhibited negative associations with P300 amplitudes located at the right frontal areas (FC6, F4 leads) which mediate inhibitory control in monitoring mechanisms [60]. Taken together these findings suggest that deductive reasoning can be cautiously described as a cascade of cognitive processes requiring concerted action of posterior areas mediating visuospatial representation and the anterior regions involved in feature integration and rule verification [61,62].

The measure of confidence was positively correlated with P300 amplitudes in the left prefrontal areas (F3 and FC5 leads) and negatively correlated in the right temporal area (T4 lead). This functional mapping seems to be in line with recent views suggesting that the remarkable ability of the human brain to adapt its information processing relies on transitory changes in patterns of cooperation and competition between neural systems encompassing specialized large-scale brain systems [61]. Additionally, given that confidence in one’s responses is a self-referential process, the Default Mode Network (DMN) may also be involved: DMN function has been linked to self-relevant, internally directed information processing [63].

Participants demonstrated decreased response accuracy as well as decreased confidence in their responses under the condition of paradoxes. Given that paradoxes involve ambiguity, the observed decrease in accuracy under that condition is compatible with reports attributing the poor outcome under ambiguous conditions to the ‘incompetence or thoughtlessness’ induced by ambiguity [63,64]. The decreased sense of confidence may also be ascribed to the ambiguity of the condition, in line with evidence suggesting that subjects feel less confident when faced with issues which they do not understand [65].

It is noteworthy that there were no correlations between behavioral performance and P300 amplitudes elicited during the condition of paradoxes. A tentative explanation of this absence of correlations under paradox processing may be the ambiguity involved in this condition, which can be reasonably expected to increase working memory load. This in turn is inversely related with P300 amplitude manifestation [58,66].

Taken together, our results suggest that deductive reasoning is subserved by several systems located both in frontal and in parietal—occipital cortices. Furthermore, it denotes that these systems are responsive to the type of deductive argument processed. Future behavioural and psychopsysiological studies might focus on understanding how the brain networks involved in reasoning are mobilised and how this mobilisation may be influenced by inter- and intra-individual factors.

*Limitations*: The main drawback of the sentence-based research practice we used in this study is the interactions between reasoning-related brain activity and higher-level linguistic processing, which constitutes a confounding factor. A second shortcoming of this experimental approach is that it neglects the fact that reasoning is often exercised on the basis of non-linguistic inputs, with information received directly from the senses. It is clearly necessary to extend the present approach to a variety of reasoning tasks, involving various administration modalities in order to ensure the generalisability of our findings.

## Conclusion

Deduction is the ability to draw necessary conclusions from previous items of knowledge. In our study the two different types of argument, paradoxical and valid, engaged different information processing operations as reflected by the involvement of frontal / orbitofrontal brain regions in dealing with paradoxical arguments, and parietal-occipital brain regions in processing valid Aristotelian arguments. The obtained results suggest that the dissociations between these two forms of brain activation in the course of reasoning are attributable to the nature of the deductive argument at hand. Paradoxical arguments implicate P3a, which originates from stimulus-driven frontal attention mechanisms during task processing. Valid arguments are associated with the P3b waveform, which originates from parietal and occipital activity and is related to attention. These comparisons elucidate similarities and differences between the neural correlates of deductive syllogistic and paradoxical reasoning. Our aim was to put forward these specific points and, in so doing, to stimulate further research into the psychophysiological basis of reasoning in general.

## Appendix

### ERP Detection with Continuous Wavelet Transform (CWT)

The CWT decomposes a signal time series, *x*(*t*), into a set of basis functions *Ψ*_*τ*,*s*_(*t*), called wavelets. Wavelets are “small waves” that grow and decay in a limited time period and have their energy concentrated in time, constituting an ideal tool for the analysis of transient, non-stationary or time-varying phenomena. As the contemporary publications indicate, CWT has a good time and frequency localization, which is ideal for ERP detection [67,68].

Wavelets are defined as [69]

$$\psi_{\tau ,s}(t)=\frac{1}{\sqrt{s}}\psi (\frac{t-\tau }{s})$$ (1)

Where $\frac{1}{\sqrt{s}}$ is a normalization parameter ensuring unit variance of the wavelet $\left\|\psi_{\tau ,s}(t)\right\|$, *τ* is the translation parameter expressing the shifting of the wavelet over the signal and *s* is the wavelet scaling parameter, which is related to the frequency. The basic functions of the wavelet transform are shifted and scaled versions of the time-localized mother wavelet, *Ψ*(*t*). For the ERP detection, the complex Morlet wavelet was chosen as a mother Wavelet.

The complex Morlet wavelet is defined as:

$$\psi (\frac{t-\tau }{s})=\frac{1}{\pi^{1/4}}e^{i\omega_{0}[(t-\tau )/s]}e^{-\frac{1}{2}{\left[(t-\tau )/s\right]}^{2}}$$ (2)

The continuous wavelet transform is the coefficient of the basis *Ψ*_*τ*,*s*_(*t*). The Morlet-Grossmann definition of the continuous wavelet transform of signal *x*(*t*) is given as [70]

Wx(τ,s)=〈x(t),ψτ,s〉=∫−∞+∞x(t)ψτ,s*(t) dt=∫−∞+∞x(t)1sψ*(t−τs) dt (3)

Using this transformation, it is possible to map a one-dimensional signal, *x*(*t*), to two-dimensional coefficients *W*_*x*(τ,s)_. The two variables can perform the time-frequency analysis, that is, the determination of a particular frequency (parameter *s*) at a certain time instant (parameter *τ*).

In our study, ERP detection was implemented by applying the following algorithm of CWT:

1. A starting and ending value of scaling (*s*) for the complex Morlet wavelet was chosen, while the translation step (*τ*) was set to an initial value of 1.
2. The correlation for the current value of scaling and for every translation was computed, covering the whole signal
3. Scaling was changed according to an appropriate step, following step 2 of the algorithm, until the maximum value of scaling had been reached

We considered the wavelet coefficients obtained by analyzing and reconstructing the evoked potential calculated via conventional averaging in each subject. A demonstration of the wavelet transform is visualized in the scalograms of Fig 2, where each coefficient represents a degree of correlation between the transformed wavelet and the analyzed signal. Based on these coefficients, an appropriate scaled wavelet was chosen to match the P300 component. The wavelet was convolved with the EEG signals, only in the corresponding part of the signal where P300 component could be situated (240–500ms after the stimulus onset), thus avoiding a false ERP detection. ERP peak values and corresponding latencies were extracted for each EEG channel of each subject, for the averaged epoched data.

## Supporting Information

S1 File Reasong material (logical statements and paradoxes used in the study). (DOCX)

S2 File Dataset of P300 amplitudes. (XLSX)
