# Supplementary material for: Aristotle Meets Zeno: Psychophysiological Evidence
Source: PLoS One. 2016 Dec 29;11(12):e0168067. doi: 10.1371/journal.pone.0168067 (PMC5199053; doi:10.1371/journal.pone.0168067)
Supplement: S1 File — (DOCX) [file pone.0168067.s001.docx]

**ARISTOTLE MEETS ZENO: PSYCHOPHYSIOLOGICAL EVIDENCE**

**SUPPORTING MATERIAL File 1: The reasoning material used**

|  | **Valid statements** | **Paradoxes** |
| --- | --- | --- |
| **1.** | All men are mortal.  All Greeks are men.  All Greeks are mortal. | Achilles and the tortoise decided to race. The tortoise started 100 meters ahead of Achilles as the latter is 100 times faster than the tortoise. Therefore, when Achilles will have advanced 100 meters the tortoise will be 1 meter ahead of him. When Achilles covers this 1 meter, the tortoise will be 1/100 meters ahead of him. When Achilles covers this 1/100 meter the tortoise will be 1/10000 meters ahead etc. Therefore, the tortoise will always be ahead of Achilles. |
| **2.** | No reptiles have fur.  All snakes are reptiles.  No snakes have fur. | A projectile will travel a given distance. This distance could be divided into two equal parts. These two parts could be divided into two equal parts. Following this rationale of continuously subdividing each part into two halves we would construct an infinite number of subparts. Thus the projectile would have to cover an infinite number of arrival/ departure courses. Given that each course would take a given amount of time, even a short one, the projectile will never reach the end of the initial distance. |
| **3.** | All kittens are playful  Some pets are kittens  Some pets are are playful | A1-A2-A3-A4-A5-A6  B1-B2-B3-B4-B5-B6  C1-C2-C3-C4-C5-C6  Arrays are equal. Array A is immobile, while arrays B and C are moving simultaneously at the same speed but in opposite direction. The target of array B is for point B1 to reach A6, the target of array C is for C1 to reach the other end of array A, point A1. Array B reaches one extreme of array A, while simultaneously array C reaches the other extreme of array A. B1 reaches one end of array A (point A6) while simultaneously C1 reaches the other end (A1). Thus B1 moved through 3A while C1 moved through 6B. That is, the time taken by one array is double of the other, although they moved for the same duration. |
| **4.** | No homework is fun.  Some reading is homework.  Some reading is not fun. | An object is at rest when it occupies a space equal to itself. A moving object occupies a place of its own size at every moment of its trajectory. Therefore, at every moment of its trajectory a moving arrow is at rest, immovable. |
| **5.** | No healthy food is fattening.  All cakes are fattening.  No cake is healthy | A Cretan claims that all Cretans are liars. |
| **6.** | All horses have hooves.  No humans have hooves.  No humans are horses. | One grain of wheat does not make a pile. Conversely, one million grains of wheat make a pile. When a given number of grains of wheat, e.g. 100 grains, do not make a pile, can we be sure that 101 grains will not make a pile of wheat? |
| **7** | No slothful person succeeds in examinations  Some pupils succeed in examinations  Some pupils are not slothful | A male barber shaves every man in town who does not shave himself but no one else. Who shaves the barber? |
| **8.** | All informative things are useful.   Some websites are not useful.  Some websites are not informative. | If a hotel with an infinite number of rooms is full, it can still take in more guests. |
| **9.** | All fruit is nutrititious  All fruit is tasty  Some tasty things nutrititious | An author writes an autobiography but he writes very slowly, so that every day of his life requires about one year of writing. If he continues at this rate, will he be able to finish this book? |
| **10.** | Some cups are beautiful  All cups are useful  Some useful things are beautiful | A covered picture is presented on a screen with the subtitle “Do you know who is covered in this picture?” “No”. “It is your father. You do not know your father!” |
| **11.** | All industrious boys in this school have red hair  Some industrious boys in this school are boarders  Some boarders in this school have red hair | The phrase "The first number not nameable in under fourteen words" appears to name it in thirteen words. |
| **12.** | All youngsters are animals  Some ravens are youngsters  Some ravens are animals | The curvature of the circumference of circle decreases as the magnitude of circle increases. For example, the curvature of the surface of earth is so negligible that it appears flat. In other words the extreme reduction of curvature is a straight line. Ergo an infinite large circle is a straight line! |
| **13.** | No jug in this cupboard is new  All jugs in this cupboard are cracked  Some cracked things in this cupboard are not new | If there is an exception to every rule, then every rule must have at least one exception. The exception to this one being that it has no exception |
| **14.** | Some cats have no tails.  All cats are mammals.  Some mammals have no tails | Several documents include pages which have the phrase: ‘This page was intentionally left blank’. Is that page blank or not? |
| **15.** | No tree is edible  Some trees are green  Some green things are not edible | "Moderation in all things, including moderation" |
| **16.** | All apples of my garden are nutritious  All nutricious fruits are ripe  Some ripe fruits in my garden are apples | It seems like you can replace any component of a ship, and it is still the same ship. So you can replace them all, one at a time, and it is still the same ship. However, you can then take all the original pieces, and assemble them into a ship. That, too, is the same ship you began with. |
| **17.** | All coloured flowers are fragnant  No fragnant flower grows indoors  No flower which grows indoors is coloured | The first number which would be regarded as more boring than interesting would get interesting due to this particular fact. |
| **18.** | Some small birds eat honey  All birds which eat honey are coloured  Some coloured birds are small | People can make decisions based not on what they actually want to do, but on what they think that other people want to do. Hence everyone decides to do something no one actually wants to do and does what he believes that everyone else wants to do. |
| **19.** | No human is perfect  All perfect creatures are mythical  Some mythical creatures are not human | How can a choice between two outcomes of exactly the same value be a rational choice? |
| **20.** | Incompetent men are men who always make mistakes  Some men who always make mistakes work here  Some men who work here are incompetent | Will anything happen if an unstoppable force strikes an immovable object? |
| **21.** | No attack on civilians is a justifiable military operation  Some military operations were attacks on civilians  Some military operations were not justifiable | Why is the night sky dark if there exists an infinity of stars, covering every part of the celestial sphere? |
| **22.** | All virtuous men have good judgment  Some of my roommate’s friends lack good judgment  Some of my roommate’s friends are not virtuous men | The existence of evil seems to be incompatible with the existence of an omnipotent, omniscient, and morally perfect God. |
| **23.** | All virtuous men possess the virtue of moderation  No one who gets drunk deliberately possesses the virtue of moderation  No one who gets drunk deliberately is a virtuous person | If all truths are recognisable, then all truths must in fact be known |
| **24.** | All Martans are inhabitants of other planets  Some Martians are not intelligent.  Some intelligent creatures are not inhabitants of other planets | You travel back in time and kill your grandfather before he conceives one of your parents, which precludes your own conception and, therefore, you couldn't go back in time and kill your grandfather. |
| **25.** | All men are capable of reasoning  No non-human creature is capable of reasoning  No non-human creature is human | If asking oneself "Am I dreaming?" in a dream proves that one is, what does it prove in waking life? |
| **26.** | Whoever corrupts the youth of his city harms himself  Socrates does not harm himself  Socrates does not corrupt the youth of his city | If truth does not exist, then the phrase “The truth does not exist” is a truth and therefore proves itself wrong. |
| **27.** | No herbivore has sharp teeth  No cow has sharp teeth  All cows are herbivores | Can an omnipotent being make a rock so heavy that even he cannot lift? |
| **28.** | No man who commits a crime unintentionally would have been prevented by the possibility of punishment  Some murderers commit crimes unintentionally  Some murderers will not be prevented by the possibility of punishment | When one pursues happiness itself, one is miserable; but, when one pursues something else, one achieves happiness. |
| **29.** | All poisons are bitter  Arsenic is not bitter  Arsenic is not poison | Water is more useful than diamonds, yet is a lot cheaper. |
| **30.** | All men who eat meat cannot declare themselves against hunting  You eat meat  Therefore, you cannot declare yourself against hunting | Increasing the price of bread makes poor people eat more of it. |
| **31.** | All mathematicians are clever  Some youths are mathematicians  Some youths are clever | Increases in production efficiency lead to even larger increases in demand. |
| **32.** | No fish is a mammal  Some creatures which live in the water are mammals  Some creatures which live in the water are not fish | If fate designed a main program which determines all that will happen, is it not useless, for example, for anyone to visit a doctor? |
| **33.** | All cats are furry mammals  Some pets are not furry mammals  Some pets are not cats | A greedy man gives away his cash with sorrow. However, he doesn't have cash with sorrow, so he gives away what he doesn't have. |
| **34.** | All the first degree murder are premeditated (deliberate/willful) homicides  No premeditated (deliberate/willful) homicide is not an act of self-defence  No act of self-defence is not first degree murder | What is better than eternal bliss? Nothing. But a slice of bread is better than nothing. So a slice of bread is better than eternal bliss. |
| **35.** | Some evergreens are subject of worship  All evergreen are trees  Some trees are subjects of worship | Answer truthfully the following question. Will the next word you say be “No”? |
| **36.** | Some snakes are not venomous  All snakes are reptiles  Some reptiles are not venomous | Can a person drown in the source of eternal life? |
| **37.** | No snakes are mammal  Some mammals are aquatic  Some aquatic creatures are not snakes | When the temperature this morning is zero degrees, and the weather forecast announces that tomorrow the temperature will double, what will be the resulting temperature? |
| **38.** | All ants are insects  Some ants have wings  Some winged creatures are insects | Your mission is not to accept this mission. Do you accept that? |
| **39.** | Some predatory birds are eagles  All eagles have exceptional sight  Some creatures with exceptional sight are predatory birds | Did you know that “0.999…” equals “1”? |
